# Priority Setting in Health Care: A Complementary Approach

**DOI:** 10.1007/s10728-013-0243-6

**Published:** 2013-03-20

**Authors:** Rui Nunes, Guilhermina Rego

**Affiliations:** Department of Bioethics, Faculty of Medicine, University of Porto, Estrada da Circunvalação 9925, 4250-150 Porto, Portugal

**Keywords:** Accountability for reasonableness, Equal opportunity function, Priorities in health care, Rationing

## Abstract

Explicit forms of rationing have already been implemented in some countries, and many of these prioritization systems resort to Norman Daniels’ “accountability for reasonableness” methodology. However, a question still remains: is “accountability for reasonableness” not only legitimate but also fair? The objective of this paper is to try to adjust “accountability for reasonableness” to the World Health Organization’s holistic view of health and propose an evolutionary perspective in relation to the “normal” functioning standard proposed by Norman Daniels. To accomplish this purpose the authors depart from the “normal” functioning standard to a model that promotes effective opportunity for everyone in health care access, because even within the “normal” functioning criteria some treatments and medical interventions should have priority upon others. Equal opportunity function is a mathematical function that helps to hierarchize moral relevant necessities in health care according to this point of view. It is concluded, first, that accountability for reasonableness is an extremely valuable tool to address the issue of setting limits in health care; second, that what is called in this paper “equal opportunity function” might reflect how accountability for reasonableness results in fair limit-setting decisions; and third, that this methodology must be further specified to best achieve fair limit-setting decisions. Indeed, when resources are especially scarce the methodology suggested in this paper might allow not only prioritizing in an “all or nothing” basis but can contribute to a hierarchy system of priorities in health care.

## Introduction

The majority of the developed countries try to associate economic growth with the provision of certain social services and, in this manner, aim at progressively improving the well-being of citizens. Accordingly, a social welfare model was developed to guarantee that everyone had, among other things, access to health care and education. However, with regards to health care a disproportionate growth of the public expenditure in this sector was verified, for various motives, especially population ageing. In 2010, 85.5 % of the health care expenditure in Norway was supported by the tax payers, in Denmark 85.1 % and in the United Kingdom 83.2 % [14]. This fact inevitably implies that limits have to be established for the public provision of health care. Also in most developed countries, namely in OECD, the proportion of total government expenditure that goes to health care is growing steadily. Indeed, in 2010 health expenditure was in Norway 9.4 % of the GDP, in Denmark 11.1 % and in the United Kingdom 9.6 % (medium of 9.5 % in OECD countries).

With the establishment of priorities in health care a more effective adjustment is possible between the demand and supply of health care. Explicit forms of rationing have already been implemented in some countries, as is the case of the Scandinavian countries, the Netherlands, England, Canada, among others, to improve the management of the considerably high public health expenditure [2]. Many of these prioritization systems resort to a proposal by Norman Daniels known as “accountability for reasonableness” [7], an indispensable tool to approach this matter.

Nevertheless, if this method provides the required legitimacy to make decisions regarding options in health care, the degree of fairness involved in these choices has been questioned. In fact, Daniels bases his proposal on two presuppositions. Firstly, that the health concept of the World Health Organization is too vast in order to achieve the appropriate choices in health care. The second presupposition, which follows from the latter, is that fairness in health care basically implies attaining a “normal” level of human performance; the citizens preferences that do not correspond to the real health requirements do not need to be met.

The objective of this study is to try to adjust the “accountability for reasonableness” to the World Health Organization’s holistic view of health and propose an evolutionary method in relation to the “normal” functioning standard proposed by Norman Daniels. Thus, the study will contribute to an even more just and equitable prioritization system in health care.

## Setting Limits in Health Care: From Legitimacy to Fairness

There are several solutions to overcome the problem of lack of sustainability in the public health care systems, though the rationalization and efficiency measures should not be neglected and should continue to be implemented. But, considering the presupposition that the possibility of increasing the contributions in the form of co-payments is very limited, the combination between efficiency and prioritization in health care has enabled the principle of equality, regarding the access to health care by everyone, to be maintained in the developed countries. Therefore, in the latter years, the establishment of priorities in health care has been considered in many western countries regarding the reform of the public health care systems. The citizens’ increasing needs, associated to ageing and the consequent demographic inversion have led to a discrepancy between demand and supply in health care.

Accountability for reasonableness is probably the most widespread model of priority setting in health care in the western world. In the United Kingdom, for instance, accountability for reasonableness helped shape thinking about how the National Institute for Health and Clinical Excellence (NICE) should incorporate social value judgments into its evidence based clinical proposals [27]. Indeed, public health systems with public accountability, such as those of Canada, the United Kingdom, New Zealand, and Sweden, are now explicitly applying this framework of accountability for reasonableness [10, 13, 17]. In the universal coverage systems of most developed countries such decisions are made by public agencies. In mixed systems, such as in the United States, decisions whether to fund new technologies—drugs, devices, procedures—are made both by public agencies and by private insurers and managed care organisations.

Although the problem of formal (democratic and public) legitimacy is usually guaranteed by accountability for reasonableness there still remains the problem of substantive legitimacy that is if justice as fairness is really considered [26].

For instance with regards the National Institute for Health and Clinical Excellence and other regulators that have direct impact on limit setting in health care it has been overtly suggested that a more inclusive process might lead us to a solution to the problem of substantive legitimacy. A decision-making process based on inclusive deliberation as an accepted standard of decision-making by the overall democratic society might add fairness to the system [30]. It follows that rational decision-making cannot be achieved on the near-exclusive basis of quantitative evidence and regulators should abide to communitarian traditions including most ethical, moral and religious traditions.

Assuming that states in a pluralistic society are at least partially neutral—in the sense that every perspective of human happiness has the same relative weight—most regulators adopt a procedural approach to ethics not a substantive one [18]. And justice is more related to fair procedures and public accountability than to any specific view of distribution of benefits and burdens. Indeed, a better framework for prioritizing health care can be achieved through the inclusion of ethical traditions that give more weight to features specific to particular persons, communities, families and political units. In a secular pluralistic society it is desirable to channel those different views of a good life resorting to democratic deliberation through democratic institutions and to the direct empowerment of the people.

Qualitative evidence will only partially allow for procedural justice in health care regulators’ deliberative process. This process in a democratic society should be objective, comparable, accountable and externally evaluated. Evidence Based Medicine (EBM) is in accordance with these principles, at a global level. This is the reason why EBM is so appealing and a determinant factor in accountability for reasonableness.

As stated by Daniels and Sabin [7], public accountability means a robust disclosure of relevant information about benefits and performance as well as a demand for a due process. A specific array of conditions should be met to comply with the principle of accountability [5–7], namely:

- Publicity condition: decisions regarding both direct and indirect limits to care and their rationales must be publicly accessible;
- Relevance condition: The rationales for limit-setting decisions should aim to provide a reasonable explanation of how the organization seeks to provide “value for money” in meeting the varied health needs for a defined population under reasonable resource constraints. Specifically a rationale will be reasonable if it appeals to evidence, reasons, and principles that are accepted as relevant by fair-minded people who are disposed to finding mutually justifiable terms of cooperation;
- Revision and appeals condition: There must be mechanisms for challenge and dispute resolution regarding limit setting decisions, and, more broadly, opportunities for revision and improvement of policies in the light of new evidence or arguments;
- Regulative condition: There is either voluntary or public regulation of the process to ensure that previous conditions are met.

This framework has as a genetic fingerprint both the deliberative process necessary to establish the legitimacy of the decision-making process as well as the fairness of such decisions [24]. Daniels claims that accountability for reasonableness makes limit-setting decisions in health care not only legitimate, but also fair. But what is really meant by fairness in limit-setting decisions?

Daniels argues that claims to equality of opportunity may be limited by scarcity of resources, but nevertheless choices and priorities in health care must be accountable by democratic procedures. This perspective of distributive justice as well as its democratic accountability is responsible for the scope and limits of health care services [5]. It follows that particular entitlements to health care—namely expensive innovative treatments and medicines—may be fairly restricted as long as this decision is socially accountable and imposed by financial restrictions of the system [23]. This framework has been used, for instance, in rationing pharmaceuticals in a accountable way [28] as the process facilitates a broader public discussion about fair limit setting [9].

The starting point of Daniel’s account of fairness [3] is that “disease and disability restricting the range of opportunities that would otherwise be open to individuals” are properly seen as unjust and not only as unfortunate circumstances. As this argument goes a right to health care access and delivery does exist as a determinant to the exercise of the equal opportunity rule. This claim for an equal opportunity of all citizens intends to ensure “normal” and not a truly “equal” functioning. This distinction seems to be paramount because each one of us is not equal to a fellow citizen in the strict sense; in fact, we are entitled to some primary goods that would allow us to function physically, psychologically and socially at a basic level. Then, our talents and capacities, following our will, may be expressed or not depending of particular circumstances. As a positive welfare right, the right to health care access and delivery imposes on society the duty to allocate resources to health related needs with maximal efficiency.

This perspective is based on a health concept distinct from the World Health Organization proposal since 1946. Concerning this aspect Norman Daniels refers that “since we believe that health is a distinct concept from happiness, we also reject the overly broad view of health as a “state of complete physical, mental, and social well-being”, and not merely the absence of disease or infirmity”, that is embodied in the World Health Organization definition [8].

By “normal” performance we mean the performance of the average citizen (criterion of the reasonable person). The “excellent” or truly “equal” performance is not the case since such a radical view of the citizens’ equal rights contradicts the proper essence of the human diversity. That is, and according to Daniels, “we correct for some “natural” effects on that distribution—disease and disability—but we do not attempt to eliminate all differences in the name of a radically expansive view of equal opportunity” [8].

The question then is how distributive decision-making in health care might gain in fairness by giving wider representation to the diversity of perspectives in contemporary western society. Specifically what is meant by “normal” functioning or behaviour is itself a matter of controversy. This is the reason why the World Health Organization definition of health is such a broad concept. On the other hand there is no hierarchy in the provision of health when the standard is “normal” versus “non-normal” functioning. It is these authors’ conviction that even in the “normal” functioning criteria some treatments and medical interventions have priority upon others.

Also, justice might be incremented with a progressive approach because in principle every health service (treatment, diagnosis, and so on) might be negotiated in the deliberative process. But fairness is also optimised because the confluence between vertical and horizontal equity implies that, even within the basic package, a progressive approach originates a true hierarchy of priorities meaning that a particular treatment has precedence over another one, although the latter may still be an integral part of the basic package.

## Equal Opportunity Function: A Complementary Approach?

**Fig. 1:**
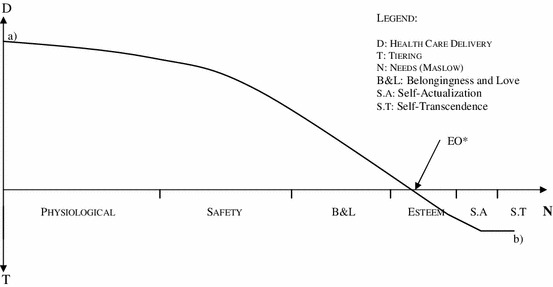
Equal opportunity function (EO)F. *D* health care delivery, *T* tiering, *N* needs (MASLOW), *B&L* belongingness and love, *S.A* self-actualization, *S.T* self-transcendence

To enable a better compatibility between “accountability for reasonableness” and the integrated view of health of the World Health Organization, the authors suggest a change of paradigm regarding the referential “all or nothing” such as proposed by Norman Daniels (“normal” vs. “non-normal” functioning) [4]. Although the logical consequence of justice as fairness being the implementation of an effective opportunity for everyone to have access to health care and, thus, the issue being the existence of a universal and general health system, its revaluation in the light of a distributive justice may dictate a different future (Fig. 1).

Equal opportunities may be represented graphically in the form of a function: (EO)F—equal opportunity function. This involves the convergence between the concepts of vertical and horizontal equity. These concepts in their philosophical and economic scope do not specify the relevant properties to characterize an agent as “equal”. This is to say, if equals should be treated in the same manner, with regards to distribution of material resources, and others in an unequal manner, it is essential to define a method which enables this distinction to be established. The graphic representation of (EO)F, in the theoretical plan, resorts to two variables:

1. The X-axis represents the variable “hierarchy of needs” in accordance with the Maslow pyramid (physiological, safety, belongingness and love, esteem, self-actualization and self-transcendence needs);
2. The Y-axis indicates the health care performance level. This variable reaches a maximum value through the universality and generality of the performance (public services) and a minimum value in which the access to health care services should be imputed to the individual responsibility of each citizen.

**Fig. 2:**
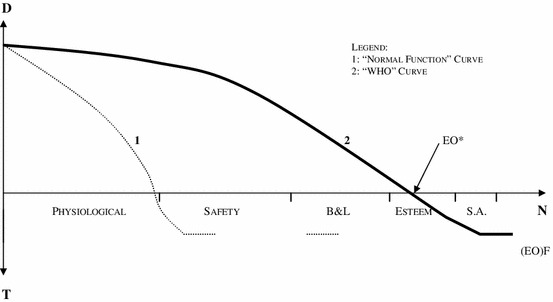
Equal opportunity function (EO)F—“normal function” version. *1* “Normal function” curve. *2* “WHO” curve

When the (EO)F intersects the X axis (Point EO*), in accordance with the combination of: a) the “needs” criteria, and b) the financial restrictions imposed by the system, appears the point from which the provision of health care through the public services is not mandatory. From this point, any citizen may have access to health care (at the cost of his own individual responsibility or the professional group which he belongs to) without damaging the principle of justice as fairness, if he resorts to alternative coverage schemes, namely private health insurance or out of pocket payments. But, only if the fulfilment of the second order needs may be at issue which may be situated, schematically, and in a variable form, as from the point that Maslow defined as esteem needs (in comparison with the “normal” functioning criteria—Fig. 2).

Norman Daniels establishes a distinction between “health need” and “health preference”, this means between the health care needs in order to reach a level of functioning—physical and psychological—typical of our species, and mere preferences, conveniences, which are beyond a reasonable and consensual level of normality. Though it is not possible to satisfy all the esteem and self-actualization needs, nor all the individual preferences, it is up to each person to resort to his financial resources (individual responsibility) to achieve this objective. As from this point EO*, and even though in an ideal society, with absolute availability of resources in which these needs could be met, in the present state of economic and financial restriction it is not only an option but, probably, a duty to proceed with this priority. In practice, and presupposing a maximum efficiency of the public health systems, the (EO)F allows the establishment of priorities in health care as far as the health care services at issue aim at meeting the needs which do not interfere with the normal performance of a citizen.

The universality of access is not subject to any objection but the claim that there are no limits to public provision (having as major criteria the individual needs, by definition, almost unlimited) is today contested since the allocation of society’s resources to health has to compete with the supply of other social goods. Indeed, one of the major challenges of modern societies is to converge economic development with human development and therefore it has been overtly suggested that a true developed society promotes every aspect of human fulfilment, such as health, education, gender equality, and so on [15].

Inevitably a delicate balance emerges between the social goods which the citizens may, or may not, be entitled to, in conformity with the social and economic situation of a particular society. And as referred by Penelope Mullen and Peter Spurgeon “the demand for health care is infinite and so rationing is inevitable”, so prioritizing has become the conceptual paradigm of the health policies of the developed countries. [20].

In fact, inequality (treating two people in a different way) becomes an inequity (treating two people in an unfair way) if a solid motive does not exist to justify the negative discrimination of a citizen in detriment of another. However, the opposite is equally valid, that is, an apparent inequity becomes a mere inequality (ethically acceptable and, therefore, fair) if it is in conformity with the restrictions imposed on the system with the application of the economic constraints in accordance with the (EO)F. But if, and only if, that situation of inequality does not harm the citizens’ basic rights and, furthermore, if the process that led to the decision is democratically determined in conformity with the public accountability criteria.

Reformulating this appreciation of the problem, the restrictions of an economic and financial nature transform a decision for allocation of resources from iniquitous (unfair) to acceptable (fair) as far as it does not negatively discriminate any class of citizens; even though a particular class of services may be legitimately restricted. This seems to be a huge dilemma of the health systems: to articulate in a fair and impartial manner the right of access to the necessary health care using the resources available in society [1, 29].

To illustrate this proposal we are going to refer to the application of equal opportunity function on elective caesarean delivery. Evidence-based medicine (EBM) has focused its attention on particular aspects of medicine, and specific areas—such as elective on-demand caesarean delivery—were left behind because there is some difficulty to perform this kind of EBM research [21]. Although there is still no absolute evidence with regards the risk/benefit analysis of on-demand caesarean delivery EBM must be carefully evaluated in this setting if adequate health policies are to be promoted [25]. Evidence should be obtained by international co-operation, by independent agencies, so that its goals are truly achieved. This evidence is necessary so that a fair resource allocation policy is accomplished. Indeed, even if it is accepted that there is a right to reproductive autonomy—allowing a woman to make informed choices about the pregnancy and birth—another issue is related to the social costs of this kind of decision. And, therefore, it should be clearly determined if it should be included in the basic health care package or if it should be left to individual responsibility.

From an ethical perspective on-demand caesarean delivery faces the problem of justice in allocation of health care resources [31]. In a changing economic environment it is not surprising that new approaches to health care may limit the provision of care as it is not possible to deliver everything to everyone. As there is no absolute evidence about the comparative risks and benefits of vaginal versus abdominal deliveries, the authors will focus specifically in the relevance condition as far as the rational and the reasons and principles for limit-setting decisions are concerned. Indeed, choices and priorities in health care are fair if they are accountable by democratic procedures.

Norman Daniels states quite clearly that “In any health care system, then, some choices will have to be made by a fair, publicly accountable, decision-making process. Just what constitutes a fair decision-making procedure for resolving moral disputes about health care entitlements is itself a matter of controversy. It is a problem that has been addressed little in the literature. Our rights are not violated, however, if the choices that are made through fair decision-making procedures turn out to be ones that do not happen to meet our personal needs, but instead meet needs of others that are judged more important” [4]. This perspective of distributive justice as well as its democratic accountability is responsible for the scope and limits of health care services. It follows that particular entitlements to health care—namely access to a particular type of delivery in a public service—may be fairly restricted as long as this decision is socially accountable and imposed by financial restrictions of the system.

In many countries, for instance, upper and middle-class women choose regularly abdominal deliveries when having babies since they can afford to pay for it in private clinics. In the private health care system, where women come from higher income and education levels, caesarean delivery prevails. On the other hand, in the public sector, where women belong to less privileged social classes, vaginal deliveries are the usual practice due to medical care routines that are imposed on them.

As far as the rational and the reasons for limit setting is concerned, and although in principle, the pregnant woman’s reproductive autonomy should be respected (see for instance the 2011 NICE’s guideline that states that “For women requesting a CS, if after discussion and offer of support … a vaginal birth is still not an acceptable option, offer a planned CS” [22]), Daniels’ relevance condition implies that a thorough economic analysis is performed. It follows that restrictions to the basic package in health care imply necessarily that the competitive medical treatment (on-demand caesarean delivery) is more expensive than the usual one (vaginal delivery). If the price is the same or lower then there is no reason based on fairness to restrict its use in public services (although it might be restricted by a risk/benefit analysis). This economic analysis should take into consideration not only direct costs but also indirect ones. In caesarean versus vaginal delivery it is usually considered only the unitary price of each procedure as determined by each country health authority (or insurance company). In general the unitary price reflects mainly the aggregated costs of the professionals, the operating room and the materials used. This analysis may be biased because the cost of a caesarean delivery is usually calculated for caesareans performed with medical indications (crash caesareans for instance) which tend to be much more expensive than caesarean on-demand, that are planned and elective.

Indeed most reports show that the cost of caesarean delivery is higher than vaginal birth. Therefore, it can be claimed that the practical application of the equal opportunity function can place caesarean delivery in the T segment (tiering) of the Y-axis that is outside the basic health care package. It follows that public health care systems have the responsibility of promoting good quality Planned Parenthood services but some interventions—such as on-demand caesarean delivery—may be legitimately positioned outside the basic package [19]. Although pregnancy and birth is in accordance with Daniels’ “normal” functioning and therefore it is a health need not a mere preference, the proposed methodology—equal opportunity function—allows that in this circumstance a fairer decision-making process is obtained. On the other hand if, in the future, it is technically demonstrated that the cost of vaginal birth is higher than that of a caesarean delivery a straightforward application of the equal opportunity function might led to the conclusion that CS might be included in the basic package. The (EO)F curve then has a great elasticity with regard the amount of services available to all citizens allowing to fairly adjust the basic package to the amount of resources available.

## Conclusion

There is no doubt that health systems must be explicit about prioritizing and Norman Daniels’ theory of justice and health is the most widespread tool to accomplish this goal. However, it faces a practical problem that is how to allocate resources fairly when they are especially scarce. Daniels claims that accountability for reasonableness makes limit-setting decisions in health care not only legitimate, but also fair.

This paper assesses the latter claim. Does accountability for reasonableness result in fair limit-setting decisions? The paper discusses different options for resolving this lack of clarity and examines how they apply to Daniels’ accountability for reasonableness framework. In general this theory holds that treatments that accomplish a species typical behaviour (normal) should be in the basic package and that this is accepted by reasonable people. And therefore a treatment that does not relate to a health need, but only to an individual preference, should be left outside [11]. Our proposal offers an evolution from this perspective. For instance pre implantation genetic diagnosis is not—strictly speaking—essential for the normal function of a person. Nevertheless, if there are enough resources available this method of diagnosis could still be offered to the general public. It would depend contingently on the balance reached by the social contract between the global amount of resources and the public services desired. It follows that the (EO)F curve is not static but slides to the left or to the right depending of particular social arrangements. It means that in principle with growing resources more health services could be in the basic package ((EO)F curve to the right) and in a period of scarcity the amount of health services is shorter ((EO)F curve to the left).

And what about prioritizing treatments that originate a species typical (normal) behaviour but that are more expensive than less expensive alternatives (such as on-demand caesarean section, some medical assisted procreation technologies, some blood alternatives or snoring surgery in non-pathological cases)? Moreover, does the traditional framework of “accountability for reasonableness” help to prioritize pharmaceuticals of proven medical benefit but extremely expensive in a just and fair way [12, 16]? Moreover, how far should society be involved in these decisions so that they are legitimate and fair [32]?

It can be claimed that applying the WHO definition of health in priority setting would allow access to a wider range of health care, without violating the four conditions underlying “accountability for reasonableness”. Indeed, publicity, revision and appeals, and regulative conditions are mainly procedural ones but are extremely useful in legitimating health care choices applying the “equal opportunity function”. On the other hand the relevance condition might even be optimized with this rationale because it can be claimed that not only a “reasonable explanation” but a “fair explanation” is given of how value for money is optimized. Namely in the European tradition equal opportunity function appeals to evidence, reasons, and principles that might be accepted as fair by most citizens. The Manchester Triage System (MTS) is a practical example of the equal opportunity function at a micro level. From a justice perspective the MTS algorithm implies that more fundamental needs (such as protecting life) have priority over less fundamental ones, although these might also be considered. At a macro level the equal opportunity function could be not only fairer but also more practical because it would also allow a more equitable managing of waiting lists, if the reasons and the rationale for choices are made public and are truly accountable.

It is concluded, first, that “accountability for reasonableness” is an extremely valuable tool to address the issue of setting limits in health care; second, that what is called “equal opportunity function” can reflect how “accountability for reasonableness” results in fair limit-setting decisions; and third, that this methodology must be further specified to best achieve fair limit-setting decisions. Indeed, when resources are especially scarce the methodology suggested in this paper will allow not only prioritizing in an “all or nothing” basis but can contribute to a more systematic and fair approach. It might even be considered as an evolutionary perspective in relation to Daniels’ account of fairness. In developed societies most citizens are aware of the need to establish priorities in health care but there is a strong disagreement about the ethical legitimacy of many choices. The “normal” functioning standard’ suggested by Norman Daniels is very appealing because the “reasonable and prudent” person can easily determine what services should be included in the basic package. But this standard may leave important modalities of diagnosis and treatment outside public services. The equal opportunity function evolves from this perspective in the sense that keeping in mind that a distinction should be made with regard the treatments that may or not be included in the basic package it allows a gradual approach and a true hierarchic system of priorities in health care.
